# Effects of personal and interpersonal factors on changes of food choices and physical activity among college students

**DOI:** 10.1371/journal.pone.0288489

**Published:** 2023-07-13

**Authors:** Juan Cao, Kun Wang, YuHui Shi, YuQing Pan, MoHan Lyu, Ying Ji, Yan Zhang

**Affiliations:** 1 Department of Social Medicine and Health Education, School of Public Health, Peking University, Beijing, China; 2 Department of Disease Control and Prevention, Peking University Third Hospital, Beijing, China; 3 Faculty of Science, The University of Sydney, Sydney, Australia; 4 Beijing Centers for Diseases Control and Prevention, Beijing, China; University of Petra (UOP), JORDAN

## Abstract

**Background:**

Health behaviors developed in the college years tend to persist in adulthood. However, distinct changing patterns of food choices and physical activity (PA) and their predictors are still less clear among college students. The current study sought to explore changes of food choices and PA, as well as the effects of personal and interpersonal factors.

**Method:**

Two-wave longitudinal data was collected from a sample of 431 Chinese college students (Mean baseline age = 19.15 ± 0.61 years; 45.7% male). A validated self-reported food frequency questionnaire was used to assess the frequency of food choices. The Chinese revised version of physical activity rating scale was used to assess physical activity. Latent profile analysis, latent transition analysis, and multinomial logistic regression analysis were used to analyze the data.

**Results:**

Two profiles of food choices, i.e., *Avoiding staples* (5.1% at Time 1) and *Varied diet* (94.9% at Time 1), were identified at both timepoints. 90.9% remained the same profiles over time, 63.6% participants in the *Avoiding staples* profile shifted to the *Varied diet* profile, and only 6.3% of those in the *Varied diet* profile shifted to the *Avoiding staples* profile. Negative body shape-related belief was related to the translation from the *Varied diet* profile to the *Avoiding staples* profile. Further, four profiles of PA, i.e., *Inactives* (51.0% at Time 1), *Low activies* (26.0% at Time 1), *Moderate activies* (15.3% at Time 1), and *Activies* (7.7% at Time 1), were identified at both timepoints. 50.8% remained the same profiles over time, 38.6% *Inactivies* shifted to the other profiles, and 48.5% *Activies* shifted to the other profiles over time. Participants with higher self-efficacy showed an increase in PA over time, and those with lower self-efficacy and lower peer support showed a decrease in PA over time.

**Conclusions:**

Overall, most of college students remained the same food choices profiles, and body shape-related belief contributed to changes in food choices profiles. About half of college students experienced changes in PA, and the predictors of such changes were peer support and self-efficacy. The findings extend the understanding of the personal and interpersonal predictors of health behaviors among college students from a dynamic perspective.

## Introduction

The college years are critical developmental period in which a large number of important transitions emerge, including leaving the parental home to attend college, increasing autonomy in decision-making, easier access to unhealthy foods, a significant reduction of academic pressure (due to their completing the most competitive college entrance examination during the period), and more social interaction [1, 2]. Since these transitions, this age period is a time when individuals’ health behaviors tend to change. Evidence from a systematic review of 49 global studies and a 10-year longitudinal study conducted in Norway suggests that there is a decline in the physical activity (PA) in late adolescence through young adulthood, compared to other age ranges [3, 4]. Further, college students leaving the parental home consumed more often high-fat/high-salt/high-sugar snacks and packaged/ready food [5]. The adverse changes in health behaviors have serious health implications, increasing the risk of obesity and noncommunicable chronic diseases (e.g., cardiovascular diseases, type 2 diabetes, respiratory diseases, musculoskeletal disorders, and various types of cancer) [6, 7], causing more psychosocial issues (e.g., lower self-esteem, higher depressive and anxiety symptoms) [8], as well as being associated with poor health throughout adulthood. Therefore, it is important to enhance understanding of factors affecting the changes of health behaviors (e.g., food choices, PA) among college students to inform the targeted healthy behaviors interventions, promote and nurture health behaviors in emerging adulthood that lay the foundations for health throughout adulthood.

Social cognitive theory (SCT), a model being widely used to interpret behavior change, indicates that individual behaviors (including health behaviors) depend on personal and interpersonal factors [9]. As college students become more independent, some personal characteristics and interpersonal influences begin to serve important roles and have specific relevance to the formation of healthy behaviors, relative to that which they served in other life stages, including: (1) psychosocial characteristics (e.g., self-efficacy) being developed or established during emerging adulthood; (2) body shape-related belief due to the greater attention to body shape [10]; and (3) peer support because of spending more time with peers and less time with family [11]. According to the SCT, self-efficacy and health belief (e.g., body shape-related belief) contribute towards overcoming the barriers to adopt and maintain health behaviors [9], with higher levels being associated with higher intakes of fruit and vegetables [12], calcium and vitamin D [13], lower intakes of high-calorie food [14], and more engagement in PA [15]. Interpersonal factors, such as peer support, are also very important in shaping individual behaviors [16]. Peer support can provide emotional, appraisal, and informational assistance to address a health-related issue for college students [17, 18]. Greater peer support for their healthy behaviors were associated with American college students’ more intake of fruit and vegetables [19], fewer intake of snacks [20], as well as Chinese college students’ more engagement in PA [21]. However, most of the relevant existing evidence was based on cross-sectional data [22], and rarely explored the associations between the above personal and interpersonal factors and health behaviors (e.g., food choices, PA) changes.

Taking together, the present study aimed to address the research question: How are the personal and interpersonal factors associated with the changes of health behaviors (i.e., food choices, PA) among college students? To address this question, drawing on two-wave (two-year interval) longitudinal data from a sample of Chinese college students, the current study: (1) explored the profiles and changes of health behaviors, and (2) examined the effects of personal (i.e., self-efficacy, body shape-related belief) and interpersonal (i.e., peer support) factors on the changes of health behaviors, accounting for sociodemographic characteristics.

## Method

### Participants

Chinese college students were recruited from a public university in Beijing, China, with more than 16000 undergraduates. A stratified cluster sampling method was adopted to acquire subjects. The sampling was taken under the stratification of academic major because of the major differences in physical activity [23], and three major academic majors (i.e., social sciences, science and engineering, and medicine) were selected. Further, cluster sampling was conducted by classes of these majors. Sample size was determined according to the general formula $n=\frac{{\left[Z_{\alpha }\sqrt{2_{p}^{-}(1{-}_{p}^{-})}+Z_{\beta }\sqrt{p_{1}(1-p_{1})+p_{2}(1-p_{2})}\right]}^{2}}{{\left(p_{2}-p_{1}\right)}^{2}}$. Here *α* = 0.05, *β* = 0.2, hence *n* = 263. Considering that the cluster sampling by class and the nonresponse rate was 10%, the sample size was increased by 0.5 times, a minimum sample size of 434 is required. A total of 451 students (freshman; 46.8% male; *M*_age_ = 19.17, *SD*_age_ = 0.63) participated in the study at Time 1 (T1, spring 2019). Of the T1 participants, 434 (3.77% attrition rate) participated in the study at Time 2 (T2, spring 2021). Attrition was mainly due to students being absent from school on the day of assessment, joining the army, or dropping out.

### Procedures

The study followed the Ethics Committees’ guidelines and was approved by the university Institutional Review Board (No. IRB00001052-19019) and the school principal of participating school. Informed consent from participants was gathered before survey data collection. Data was collected through self-administered electronic web-based questionnaires. The link to the survey was posted on and circulated through various social media platforms. Participants were informed to withdraw from the study at any time if they do not wish to participate.

### Measures

#### Independent variable

*Sociodemographic variables*. Gender (0 = male, 1 = female), residence (0 = urban areas, 1 = rural areas), parental education level (ranging from 1 = uneducated to 7 = master degree or above), and BMI at T1 were considered when examining predictors of changes, given their impact on health behaviors of college students [24, 25].

*Self-efficacy (Time 1)*. Self-efficacy reflects a person’s belief in his or her ability to overcome the difficulties inherent in performing a specific task in a particular situation [26]. The difficulties related to healthy eating and physical activity include internal (e.g., physical functioning, psychological conditions) and external difficulties (e.g., lack of facilities, environmental conditions) [27]. Further, prior study has shown the importance of matching domain-specific self-efficacy to the type of behavior being investigation [28]. Thus, the study focused on domain-specific self-efficacy, i.e., diet self-efficacy and PA self-efficacy. Participants were asked to answer questions about how hard it would be to engage in behaviors related to diet/PA on a 5-point scale ranging from 1 (strongly disagree) to 5 (strongly agree), with higher scores reflecting higher diet/PA self-efficacy. Before participants answered the diet self-efficacy subscale, the definition of healthy diet based on the Chinese Dietary Guidelines (i.e., higher frequency for intaking staples, vegetables, fruits, and protein intake, as well as less intake of sweets/sugary drinks/snack foods) has been provided for participants [29]. The diet self-efficacy subscale includes five items, including “I have the confidence to stick to a healthy diet”, “Even when environmental conditions or other factors are not friendly, I still have ways to stick to a healthy diet”, “To maintain a healthy body or body shape, I can stick to a healthy diet”, “I can restrain myself from eating unhealthy food and maintain a healthy diet”, and “I have a healthy eating plan and can choose the foods I eat according to the plan”. PA self-efficacy subscale includes five items, including “I have confidence that I can keep doing PA”, “Even if the environmental conditions or other factors are not friendly (e.g., the bad weather), I will engage in PA in other ways”, “To maintain healthy body or body shape, I can keep PA”, “I was able to overcome subjective experiences (e.g., laziness, tiredness) and keep doing PA”, “I have my own PA plan and I can follow it”. Confirmatory factor analysis for diet self-efficacy subscale indicated that the one-factor model demonstrated a good fit to the data: χ^2^/*df* = 10.60, CFI = 0.97, TLI = 0.93, SRMR = 0.03. Confirmatory factor analysis for PA self-efficacy subscale also indicated that the one-factor model demonstrated a good fit to the data: χ^2^/*df* = 3.72, CFI = 0.99, TLI = 0.98, SRMR = 0.02. The results indicated that diet self-efficacy and PA self-efficacy subscales showed good construct validity. The Cronbach’s alphas for diet self-efficacy and PA self-efficacy subscales were 0.91 and 0.90, respectively.

*Body shape-related belief (Time 1)*. Four items were used to assess the participants’ body shape-related belief on a 5 Likert scale ranging from 1 (strongly agree) to 5 (strongly disagree), with higher scores reflecting more positive belief related to body shape. Items include “I’m satisfied with my current body shape” (reverse scoring), “I get out of my shape easily”, “Not being in ideal shape is hard for me to accept”, and “I hope to change my current body shape”. Confirmatory factor analysis indicated that the one-factor model demonstrated a good fit to the data: χ^2^/*df* = 5.13, CFI = 0.98, TLI = 0.95, SRMR = 0.03. The results indicated that this scale showed good construct validity. The Cronbach’s alpha for this scale was 0.73.

*Peer support (Time 1)*. Participants’ perceived peer supports for healthy diet and PA were assessed on a 5 Likert scale ranging from 1 (strongly disagree) to 5 (strongly agree), with higher scores reflecting higher levels of peer support. Peer support for healthy diet subscale includes seven items, including “My friends encouraged me to adopt a healthy diet”, “My friends often reminded me to adopt a healthy diet”, “My friends joined me in adopting a healthy diet”, “Adopting healthy diet will get praise from friends”, “My friends restricted me to eat sugary foods/drinks/snack foods”, “My friends joined me in eating sugary foods/drinks/snack foods” (reverse scoring), and “Eat less sugary foods/drinks/snack foods will get praise from friends”. Peer support for PA subscale includes five items, including “My friends encouraged me to engage in PA”, “My friends often reminded me to engage in PA”, “My friends joined me in engaging in PA”, “Engaging in PA will get praise from friends”, and “My friends will be willing to buy or share exercise equipment and gym memberships with me”. Confirmatory factor analysis for peer support for healthy diet subscale indicated that the one-factor model demonstrated a good fit to the data: χ^2^/*df* = 3.66, CFI = 0.98, TLI = 0.97, SRMR = 0.03. Confirmatory factor analysis for peer support for PA subscale also indicated that the one-factor model demonstrated a good fit to the data: χ^2^/*df* = 10.38, CFI = 0.95, TLI = 0.90, SRMR = 0.04. The results indicated that peer support for healthy diet and PA subscales showed good construct validity. The Cronbach’s alphas for peer support for healthy diet and PA subscales were 0.84 and 0.84, respectively.

#### Dependent variables

*Food choices (Time 1*, *Time 2)*. Participants were asked to describe the frequency of food choices during the past 30 days on a 5-point scale (1 = never, 2 = 1 day or less per week, 3 = 2–3 days per week, 4 = 4–5 days per week, 5 = almost every day). According to the Chinese Dietary Guidelines [29], five kinds of food group were included: staples (including cereals, tubers), fruits and vegetables (including dark-green leafy vegetables, vitamin A-rich fruits and vegetables, other fruits and vegetables), meat (including pork/beef/mutton livestock meat, chicken/duck/goose poultry meat, aquatic products); legumes (including bean curd, soybean milk), eggs and dairy products (including milk, yoghurt, and other dairy products). Further, given that Chinese college students tend to have particularly high sugar-sweetened food (including sweetened chocolate, and pastries) consumption rates, sweets were included in the analysis for food choices patterns.

*Physical activity (Time 1*, *Time 2)*. In the study, PA was defined as self-disciplined, planned, and repetitive PA with a certain intensity, frequency, and duration. PA was measured using the Chinese revised version of the Physical Activity Rating Scale [30]. Participants were asked to complete the information about PA during the past one month (Time 1, spring 2019, the first year of college) and the past one month (Time 2, spring 2021, the third year of college). Items included: 1) Intensity: did you conduct light (e.g., walking, doing radio exercises)/low (e.g., jogging, doing Tai Chi)/moderate (e.g., running, play ping pong)/vigorous (e.g., badminton, basketball, tennis, race, aerobics) activities; 2) Duration: if yes, participants was asked to rate how much time you spent on PA each time on a 4-point scale, including 1 (≤10min), 2 (11-30min), 3 (31-60min), and 4 (≥60min); and 3) Frequency: participants was asked to rate how often you spent on PA on a 6-point scale, including 1(one time/month), 2 (2–3 times/month), 3 (one time/week), 4 (2–3 times/week), 5 (4–5 times/week), and 6 (almost once a day). The score of light PA = ∑*duration*×*frequency*, and so on for the scores of other intensity PA. The overall score of physical activity = ∑*intensity*×*duration*×*frequency*. Previous studies showed that the scale demonstrated adequate reliability and validity in the Chinese population [31].

#### Data analytic strategy

All analyses were conducted using M*plus* Version 7.0 in the following steps [32]. First, latent profile analysis (LPA) was conducted to identify distinct profiles for food choices and PA at Time 1 and Time 2 separately [33]. Models specifying 1 to 5 profiles were initially tested. To determine the relative fit of the models, we compared models based on the following statistical fit indices: (1): Akaike information criteria (AIC) and Sample Size Adjusted Bayesian Information Criterion (aBIC) scores, with the lower values indicating a better fitting model; (2) Lo–Mendell–Rubin(LMR) and Bootstrapped Lo–Mendell–Rubin likelihood ratio test (BLRT), with significant *p* values indicating that the model with k profiles is a better fit of the data than the model with k-1 profiles; and (3) each class size is larger than 5% to ensure better generalizability [34]. LPA, which is one of person-centered approach, have advantages over traditional variable-centered approach (e.g., like regression, factor analysis). Rather than grouping similar items and variables, LPA provide a way of grouping individuals into categories based on shared characteristics that distinguish members of one group from members of another group [35]. The method was often used in prior studies examining dietary patterns (based on frequency of food consumption) and their predictors [36, 37]. Second, based on the profiles extracted from the LPAs, an unconditional latent transition analysis was built to determine changes in food choices and PA over time via estimating transition probabilities [38]. Third, to examine the contributions of personal (i.e., self-efficacy, body shape-related belief) and interpersonal (i.e., peer support) factors in predicting the changes of food choices and PA, the current study conducted multinomial logistic regression analysis using changes as dependent variables, with self-efficacy, body shape-related belief, and peer support as the predictor variables, accounting for sociodemographic characteristics (i.e., gender, residence, parental education level, and BMI).

## Results

For the purpose of the study, the final analytical sample included 431students (45.7% male; Mean baseline age = 19.15 ± 0.61 years), who responded to measures of food choices both at T1 and T2. Comparisons among participants who participated in the T1 assessment and those who participated in both T1 and T2 assessment indicated no significant differences in all studied variables and sociodemographic variables (*p* > 0.05). These results indicated that the missingness was random [39].

Most of participants reported that their residence was urban (82.1%) and had normal body mass index (77.5%). More than 50% of the participants reported that their parents had college and above. The whole sociodemographic information at T1, such as gender, residence, parental education level, and BMI, are presented in Table 1.

**Table 1:** Demographic characteristics at T1 of participants (*N* = 431).

3000		Frequency	Percent (%)
Gender			
	Female	234	54.3
	Male	197	45.7
Residence			
	Urban	354	82.1
	Rural	77	17.9
Parental education			
	Middle school and below	66	15.3
	High school	75	17.4
	Associated degree	66	15.3
	College and above	224	52
BMI (kg.m^-2^)			
	< 18.5	52	12.1
	18.5–24.9	334	77.5
	≥25	45	10.4

### Profiles and changes

For each time point, independent latent profile analyses for food choices and PA were conducted, beginning with a one- profile model and fitting up to a five- profile model.

### Food choices

Latent profile analysis examined profiles of food choices using six indicators (i.e., staples, vegetables/fruits, dairy products/eggs, meat, legumes, and sweets). Examination of these results revealed that a two-profile solution was determined to be the most appropriate for the T1 and T2 data. The model fit indices associated with the cross-sectional latent profile analysis were estimated at each time point and reported in Table 2. Model fit statistics for T1 and T2, including: (1) significant *p* values of LMR and BLRT for a two-profile model; (2) relatively reductions in AIC, BIC, and adjusted BIC values by adding the third profile; (3) lower entropy value for the three, four, and five-profile model; and (4) very small numbers of participants classified into the fourth and fifth profiles (<5%), suggested an optimal two-profile solution both for T1 and T2.

**Table 2:** Fit statistics for latent profiles models of food choice at Time 1 and Time 2.

	AIC	BIC	aBIC	Entropy	LMR (*p* value)	BLRT (*p* value)	SC n (%)
T1							
C1	6478.33	6527.12	6489.04	—	—	—	—
** C2**	**5907.75**	**5985.01**	**5924.71**	**0.999**	**0.018**	**<0.001**	**22 (5.10)**
C3	5662.55	5768.27	5685.76	0.991	0.117	<0.001	22 (5.10)
C4	4809.81	4943.99	4839.27	0.998	0.815	<0.001	7 (1.62)
C5	5369.51	5532.14	5405.21	0.811	0.151	<0.001	7 (1.62)
T2							
C1	6500.71	6549.51	6511.43	—	—	—	—
** C2**	**6066.36**	**6143.61**	**6083.32**	**0.998**	**<0.001**	**<0.001**	**33 (7.66)**
C3	5861.01	5966.72	5884.21	0.968	0.308	<0.001	32 (7.43)
C4	5593.36	5727.54	5622.82	0.875	0.285	<0.001	31 (7.19)
C5	5392.52	5555.15	5428.22	0.887	0.141	<0.001	13 (3.02)

*Note*. The optimal solution is bolded. Smaller values of AIC and BIC indicate better model fit. A high value of entropy (>0.80, closer to 1) reflects a more accurate classification. Significant p-value of LMR and BLRT indicate improvement of k classes over k-1 classes [38]. Each class size is larger than 5% to ensure better generalizability. AIC = Akaike information criteria; BIC = Bayesian information criteria; BLRT = Bootstrapped Likelihood Ratio Test; SC = Smallest class size.

**Fig 1:**
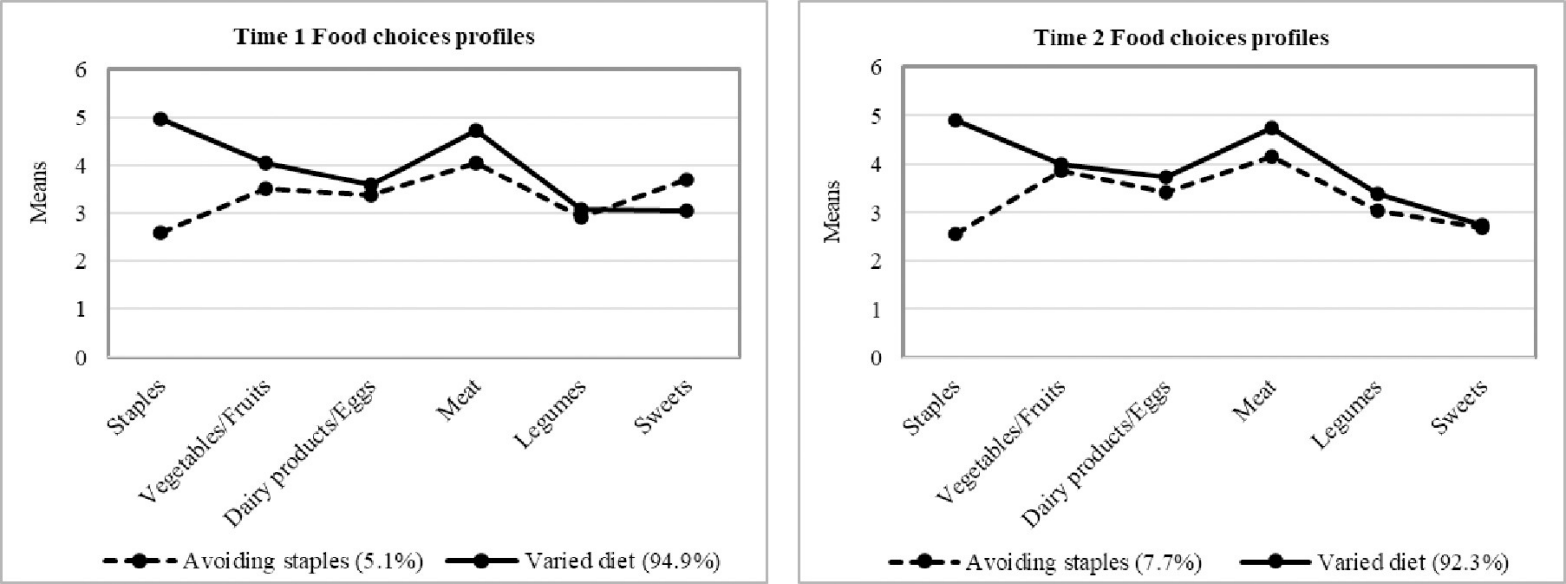
Two food choices profiles at Time 1 and Time 2.

Fig 1 showed the mean scores for each of the two profiles of food choices at T1 and T2. Specifically, the following labels were offered for the two emerging profiles at T1: *Avoiding staples* (i.e., lower frequency of choosing staples than other types of food; 5.1%) and *Varied diet* (i.e., lower frequency for choosing high-sugar food but higher frequency for choosing all other kinds of food; 94.9%). And, the labels for the two profiles at T2 also included: *Avoiding staples* (7.7%) and *Varied diet* (92.3%).

**Fig 2:**
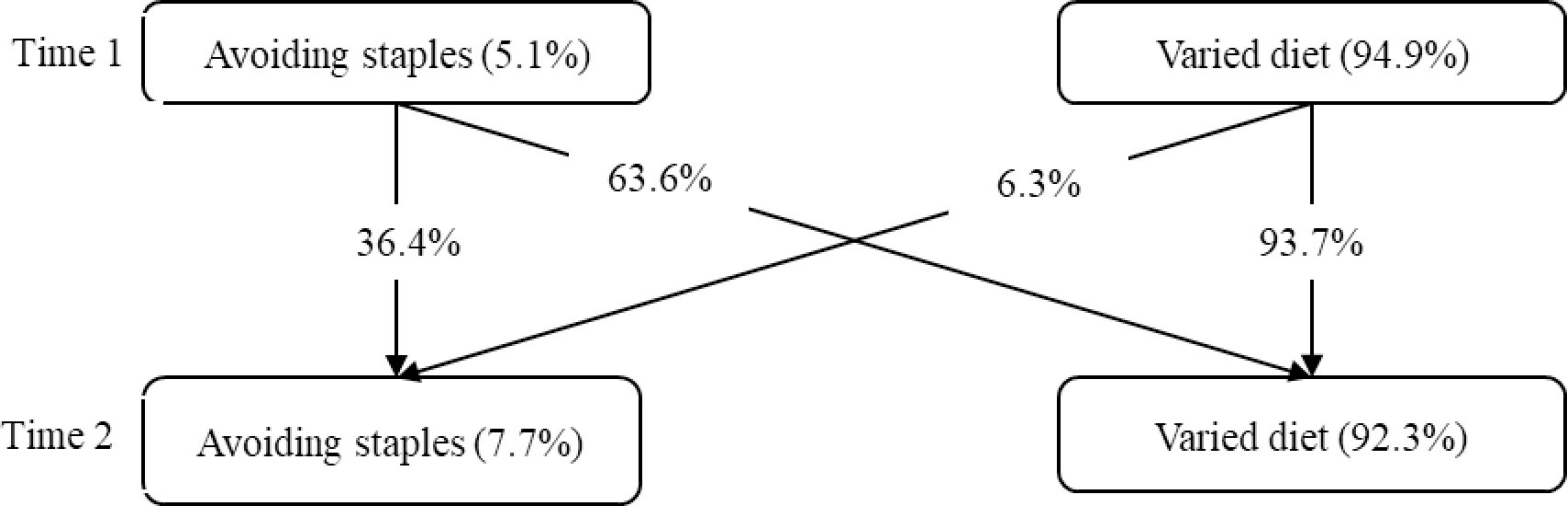
Unconditioned latent transition probability of food choices profiles.

Stability and change in different profiles of food choices over two years were shown in Fig 2. The *Varied diet* was rather stable over two years. Those in the *Varied diet* group at T1 had a high probability of 93.7% still being classified as the same group at T2, while they only had a probability of 6.3% shifting to the *Avoiding staples* pattern at T2. The *Avoiding staples* exhibited instability across T1 to T2, with 63.3% of the group shifted to the *Varied diet* group over time.

### Physical activity

Latent profile analysis examined profiles of physical activity using five indicators (i.e., light PA, low PA, moderate PA, vigorous PA, and overall PA). Examination of these results revealed that a four-profile solution was determined to be the most appropriate for the T1 and T2 data. Model fit statistics for T1 and T2 (Table 3), including: (1) significant *p* values of LMR and BLRT for a four-profile model; (2) relatively reductions in AIC, BIC, and adjusted BIC values by adding the fifth profile, suggested an optimal four-profile solution both for T1 and T2.

**Table 3:** Fit statistics for latent profiles models of physical activity at Time 1 and Time 2.

	AIC	BIC	aBIC	Entropy	LMR (*p* value)	BLRT (*p* value)	SC n (%)
T1							
C1	6130.62	6171.28	6139.55	—	—	—	—
C2	5451.81	5516.86	5466.09	0.979	<0.001	<0.001	87 (20.19)
C3	5225.66	5315.11	5245.29	0.925	0.035	<0.001	84 (19.49)
**C4**	**4893.49**	**5007.35**	**4918.49**	**0.945**	**0.027**	**<0.001**	**33 (7.66)**
C5	4747.21	4885.46	4777.56	0.971	0.051	<0.001	15 (3.48)
T2							
C1	6130.62	6171.28	6139.55	—	—	—	—
C2	5519.72	5584.77	5534.01	0.959	<0.001	<0.001	93 (21.60)
C3	5200.21	5289.66	5219.84	0.929	0.002	<0.001	85 (19.72)
**C4**	**4883.61**	**4997.45**	**4908.61**	**0.961**	**<0.001**	**<0.001**	**37 (8.59)**
C5	4682.46	4820.71	4712.81	0.981	0.145	<0.001	32 (7.52)

*Note*. The optimal solution is bolded. AIC = Akaike information criteria; BIC = Bayesian information criteria; BLRT = Bootstrapped Likelihood Ratio Test; SC = Smallest class size.

**Fig 3:**
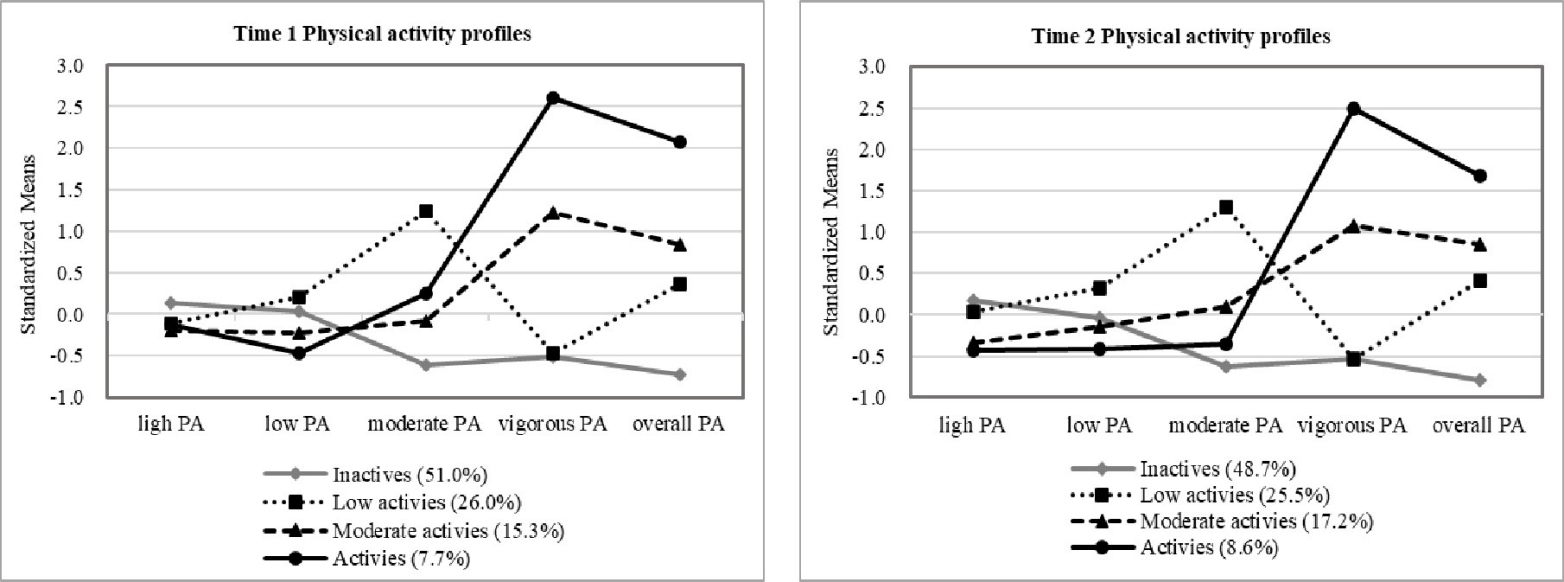
Four physical activity profiles at Time 1 and Time 2.

Fig 3 showed the standardized means for each of the four profiles of PA at T1 and T2. Specifically, the following labels were offered for the four profiles at T1: *Inactives* (i.e., the lowest levels of moderate, vigorous, and overall PA; 51.0%), *Low activies* (i.e., the highest levels of moderate PA and relatively lower levels of overall PA; 26.0%), *Moderate activies* (i.e., the higher levels of vigorous and overall PA; 15.3%), and *Activies* (i.e., the highest levels of vigorous and overall PA; 7.7%). And, the labels for the four profiles at T2 also included: *Inactives* (48.7%), *Low activies* (25.5%), *Moderate activies* (17.2%), and *Activies* (8.6%).

**Fig 4:**
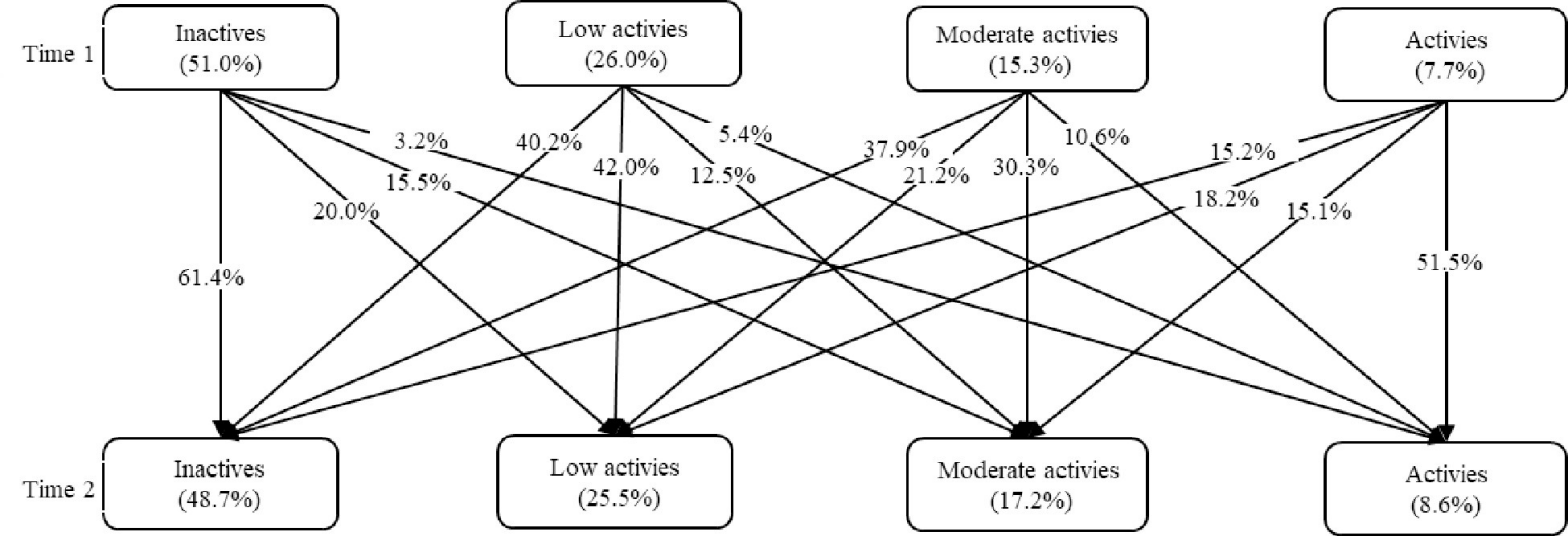
Unconditioned latent transition probability of physical activity profiles.

Stability and change in different profiles of PA over two years were shown in Fig 4. *Inactives* were relatively stable, with 61.4% still being classified as the same group over time. Nearly 50% of participants in *Activies* group engaged less PA over time, with shifting to the *Inactives* (15.2%), *Low activies* (18.2%), *Moderate activies* (15.1%) groups at T2, respectively.

### Predictors of changes

Multinomial logistic regression for the changes of food choices and PA were independently conducted to determine whether personal (i.e., self-efficacy, body shape-related belief) and interpersonal (i.e., peer support) characteristics were predictive of changes in PA over time, accounting for sociodemographic characteristics (i.e., gender, parental education level, and BMI).

### Food choices

Table 4 includes odds ratios (ORs) for T1 predictors of changes in food choices from T1 to T2. For sociodemographic characteristics, gender was the only significant factor of predicting changes in food choices. Specifically, girls had significantly higher odds than boys of transferring from the *Varied diet* group at T1 to the *Avoiding staples* group at T2 (*OR* = 4.22, *p* = 0.018; relative to those remaining in the *Varied diet* group). As for personal characteristics, body shape-related belief was the only significant predictor of the changes of food choices. Specifically, more negative body shape-related belief at T1 was associated with higher odds of transferring from the *Varied diet* group to the *Avoiding staples* group (*OR* = 0.41, *p* < 0.001) than remaining in the *Varied diet* group, indicating lower frequency of choosing staples among participants with negative body shape-related belief. However, the effects of self-efficacy and peer support on the changes of food choices were insignificant.

**Table 4:** Odds ratios for covariates predicting changes of food choices.

Predictors	Time 1 Food choices profiles	Time 2 Food choices profiles	
		*Avoiding staples*	*Varied diet*
		*OR* [95%CI]	*OR [95%CI]*
Gender	*Avoiding staples*	ref	0.43 [0.04, 5.06]
	*Varied diet*	**4.22 [1.28, 13.94]** ^*^	ref
Residence	*Avoiding staples*	ref	1.91 [0.16, 22.20]
	*Varied diet*	1.14 [0.41, 3.15]	ref
Parental education levels	*Avoiding staples*	ref	0.90 [0.47, 1.74]
	*Varied diet*	1.01 [0.75, 1.35]	ref
BMI	*Avoiding staples*	ref	1.32 [0.85, 2.06]
	*Varied diet*	0.98 [0.84, 1.13]	ref
Self-efficacy	*Avoiding staples*	ref	0.49 [0.20, 1.16]
	*Varied diet*	0.73 [0.47, 1.12]	ref
Body shape-based belief	*Avoiding staples*	ref	0.88 [0.31, 2.47]
	*Varied diet*	**0.41 [0.24, 0.69]** ^***^	ref
Peer support	*Avoiding staples*	ref	0.73 [0.28, 1.88]
	*Varied diet*	1.07 [0.69, 1.66]	**ref**

*Note*. Ref = Reference class; gender coded as 0 = boy and 1 = girl; Residence coded as 0 = urban and 1 = rural

*p < 0.05

***p < 0.001.

### Physical activity

Table 5 includes odds ratios (ORs) for T1 predictors of changes in PA from T1 to T2. For sociodemographic characteristics, college students whose parents had higher education levels were more likely to transfer from *Inactives* to *Low activies* (*OR* = 1.38, *p* = 0.014) and *Moderate activies* (*OR* = 1.67, *p* < 0.001) over time. Higher level of self-efficacy significantly increased the odds of transferring from *Inactives* to *Low activies* (*OR* = 1.63, *p* = 0.026) and *Activies* (*OR* = 4.45, *p* = 0.004) over time; and lower self-efficacy significantly increased the odds of transferring from *Activies* to *Inactives* (*OR* = 0.11, *p* = 0.002) and *Moderate activies* (*OR* = 0.17, *p* = 0.009) over time. Further, lower peer support significantly increased the odds of transferring from *Activies* to *Low activies* (*OR* = 0.07, *p* = 0.006), relative to remaining in the *Activies* group. These results indicated an increase in physical activity among participants with higher self-efficacy and a reduction in physical activity among participants with lower self-efficacy and lower peer support. However, the effect of body shape-related belief on the changes of PA was insignificant.

**Table 5:** Odds ratios for covariates predicting changes of physical activity.

		Time 2 Physical activity profiles			
Predictors	Time 1 Physical activity profiles	*Inactives*	*Low activies*	*Moderate activies*	*Activies*
		*OR [95%CI]*	*OR [95%CI]*	*OR [95%CI]*	*OR [95%CI]*
Gender	*Inactives*	ref	0.67 [0.32, 1.41]	1.18 [0.49, 2.84]	0.68 [0.13, 3.49]
	*Activies*	1.80 [0.21, 15.10]	0.56 [0.07, 4.70]	0.15 [0.01, 1.73]	ref
Residence	*Inactives*	ref	0.58 [0.22, 1.50]	0.49 [0.16, 1.50]	0.32 [0.07, 1.43]
	*Activies*	1.88 [1.13, 26.32]	1.50 [0.11, 20.30]	1.88 [0.13, 26.32]	ref
PEL	*Inactives*	ref	**1.38 [1.07, 1.79]** *	**1.67 [1.22, 2.28]** ***	1.70 [0.88, 3.27]
	*Activies*	1.06 [0.43, 2.60]	0.70 [0.31, 1.59]	2.63 [0.91, 7.61]	ref
BMI	*Inactives*	ref	1.09 [0.99, 1.19]	1.04 [0.92, 1.18]	1.06 [0.86, 1.30]
	*Activies*	1.01 [0.84, 1.21]	0.87 [0.63, 1.21]	0.84 [0.62, 1.14]	ref
SE	*Inactives*	ref	**1.63 [1.06, 2.51]** *	1.10 [0.70, 1.74]	**4.45 [1.61, 12.28]** **
	*Activies*	**0.11 [0.03, 0.44]** **	1.17 [.89, 1.54]	**0.17 [0.05, 0.64]** **	ref
BSB	*Inactives*	ref	0.78 [0.52, 1.17]	1.17 [0.69, 1.98]	0.97 [0.40, 2.33]
	*Activies*	1.31 [0.44, 3.92]	.89 [.68, 1.18]	1.58 [0.53, 4.68]	ref
PS	*Inactives*	ref	0.93 [0.63, 1.36]	0.64 [0.41, 1.01]	1.18 [0.53, 2.63]
	*Activies*	0.36 [0.11, 1.14]	**0.07 [0.01, 0.47]** **	0.54 [0.18, 1.62]	ref

*Note*. PEL = Parental education level; SE = Self-efficacy; BSB = Body shape-based belief; PS = Peer support, Ref = Reference class; Gender coded as 0 = boy and 1 = girl; Residence coded as 0 = urban and 1 = rural

*p < 0.05

**p < 0.01

***p < 0.001.

## Discussion

Using a longitudinal design, the present study identified the profiles and changes of food choices and PA and examined specific personal (i.e., self-efficacy, body shape-related belief) and interpersonal (i.e., peer support) predictors among college students. The findings revealed two profiles in food choices and four profiles in PA both at T1 and T2 (food choices: *Avoiding staples*, *Varied diet*; PA: *Inactives*, *Low activies*, *Moderate activies*, and *Activies*). Most participants in *Avoiding staples* group shifted to the *Varied diet* group, and nearly 50% *Activies* shifted to the other three groups over time. Furthermore, the study also uncovered the importance of self-efficacy, body shape-related belief, and peer support on food choices and PA changes, which may help inform targeted intervention and prevention programs.

### Changes

Although prior study demonstrated that dietary habits established in the first year of college likely carry forward into later college years [40], this study contributes uniquely to the literature by unpacking a stability tendency for *Varied diet* and a predominant change toward *Varied diet* from *Avoiding staples* among college students. Regular staples intake increases dietary fibre intake and decrease the problem of constipation; thus, infrequent intake of staples is discouraged by parents, especially in China. College students are more likely to change their behaviors when the behaviors may be discouraged by significant others [41], which may explain an instability in *Avoiding staples*. Further, although behavior patterns can be disrupted as individuals adapt to new circumstances (e.g., entering college), most of college students (93.7%) in *Varied diet* group generally remained in the same group over time. The finding indicated that varied food choices pattern (defined as a mix of food choices) may become ingrained during the university.

Despite a general declining tendency in PA among college students [42], this study used a novel person-centered statistical technique to explore changes of PA during the university and found that the evolution of PA varied greatly. Specifically, most (61.4%) *Inactives* and over 50% *Activies* remained in the same group, and nearly 50% *Activies* shifted to the other three groups over time. These findings indicated either a stable (i.e., consistently-inactive/active) or a declining tendency in PA of college students, which are consistent with an observation on three predominant trajectories of PA (*Inactivity maintainers*, *Activity maintainers*, *Decreasers from moderate PA*) in adolescence through young adulthood [43]. However, this study did not provide support for the habit formation hypothesis suggesting that physical activity behaviors become automatic once it has been repeatedly practiced over time [44]. It is possible that engaging in physical activity is more likely to be disrupted by other factors (such as lack of time, accessible and convenient space, as well as equipment for physical activity), relative to food choices. In a survey about perceived barriers to keeping physical activity in college students, the biggest deterrent to exercise habits was “lack of time” (36%) [45]. It seems likely that the changes of PA may provide opportunities for effective intervention [46]. However, there are also some challenges in PA intervention for college students, including: how to prevent a drop in PA, how to support the maintenance of high PA, and how to promote PA.

### Predictors of changes

Another contribution of this study is the examination of the influences of personal (i.e., self-efficacy, body shape-related belief) and interpersonal predictors (i.e., peer support) factors on changes of food choices and PA to develop a preliminary understanding of the origins of the patterns and the subsequent transitions as well as to further inform assessment and intervention efforts. Results observed that the predictors of changes of food choices was different from that of physical activity. Specifically, more negative body shape-related belief was associated with higher odds of transferring from the *Varied diet* group to the *Avoiding staples* group, while higher self-efficacy was related to an increase in physical activity and lower self-efficacy and peer support were related to a reduction in physical activity. This finding might reveal a different influencing mechanism between the two health behaviors changes, and support the use of tailored intervention for the different health behaviors.

Body shape-related belief reflects the subjective perception and the degree of satisfaction about one’s own body shape, with lower score representing that a negative perception and a dissatisfaction about their own body shape. Previous study observed that the majority (81%) of college students were dissatisfied with their body shape [47]. When college students feel dissatisfied with their body shape, they are more likely to reduce their intake of high-fat and -calorie food (e.g., staples) to manage it, rather than other behaviors (e.g., engaging in physical activity) [47]. Thus, body shape-related belief is an important predictive factor in food choices changes, which could inform practitioners to enhance the knowledge and skills in weight management in order to achieve healthy eating behavior.

It should be noted that the significant effects of diet self-efficacy on food choices changes were not observed in this study. This finding is inconsistent with the health belief model, indicating the association between self-efficacy and nutrition behavior [48]. This difference in results reflects the notion that individuals are not influenced by self-efficacy to an equal extent, and the associations between self-efficacy and food choices changes may be affected by other factors (e.g., nutrition knowledge). Nutrition knowledge can empower students to make informed dietary choices [49], and greater diet self-efficacy was associated with more frequent intake of healthy foods only in college students with nutrition knowledge [50]. Further, from a sample of college students, a cross-sectional study found that self-efficacy did not directly predict eating behavior, but was a predictor of intention to reduce fat intake [51]. It would be informative for future research to examine the indirect assosication between self-efficacy and food choices changes. Conversely, PA self-efficacy has been shown to be a predictor of the changes of PA, indicating an increase in PA among participants with higher PA self-efficacy and a reduction in PA among participants with lower PA self-efficacy. This finding is in line with prior research documenting the PA self-efficacy’s strong prediction of PA [52]. Social learning theory indicated that individuals with higher PA self-efficacy are more likely to view obstacles as challenges, instead as barriers, which may explain the positive contribution of higher self-efficacy to the improvement of PA [53]. However, college students who perceived low self-efficacy for PA were less physically active over time, since impaired thought patterns and stress reactions caused by low self-efficacy may create internal barriers to engaging in PA [26]. This study extends the dearth of research examining the associations between self-efficacy and health behaviors changes of college students.

Further, during emerging adulthood, students spend considerable time with peers and become more independent of parents, peer influences play a significant role in health behaviors, such as providing motivation and help in making for healthy behaviors choices [54]. However, the study observed that there were significant associations between peer support and PA changes but not between peer support and food choices changes. The findings were consistent with prior studies indicating peers’ influence to be the least important motivator for varied diet [55], and a positive link between peer support and sufficient PA among college students [15], highlighting the notion that it is important to involve college students’ wider network of peers in promoting their PA. One explanation may be related to the developmental characteristics of emerging adulthood. The period is recognized as a time when individuals seek peer approval and social identity. As such, college students may want to gain social affiliation and competitiveness, which were the main motivating factors for college students’ engagement in PA [56].

### Limitations

Several limitations should be noted when interpreting the findings. First, all data were collected with self-reports, which could yield a threat to internal validity. Gathering data from multiple sources (e.g., friends and parents), observation or official reports should be useful in future research to increase confidence in the measurements by reducing the effects of common method bias and overly positive self-presentation. Second, the results are constrained by the limited representativeness of the sample which was recruited solely from a single university in China. Thus, caution should be used in generalizing the results from the present study to college students from other universities. It would be informative for future research to sample a larger group of college students with more regionally and socioeconomically diverse backgrounds to examine the nuances of food choices and PA changes, and use bigger samples for sub-group comparisons including males and females, or younger and older participants. Third, the profiles of food choices were identified based on the intake frequency (not quantity) of different kinds of food. Future research could consider food quantity to provide more comprehensive information to assess food choices changes. Fourth, the latent transition analysis was performed without controlling for the effects that developmental changes (e.g., stressors) during emerging adulthood may have on health behaviors changes. Examples of such stressors that would be beneficial to include in future studies could lie in the interpersonal domain, relating to the school environment (a transition from high school to college students), peer relationships (the emergence of romantic relationships), individual factors (physiological changes), and leaving home.
